# Predictors of quality of life and mental health in breast cancer survivors in Northern Iran

**DOI:** 10.1186/s12905-023-02533-7

**Published:** 2023-07-18

**Authors:** Fatemeh Zolfaghary, Reza MashaghiTabari, Mobina Dezhman, Ali Bijani, Farzan Kheirkha, Hajar Adib-Rad

**Affiliations:** 1 Student Research Committee, Babol University of Medical Sciences, Babol, Iran; 2 Faculty of Medicine, Belgrade University of Medical Sciences, Belgrade, Serbia; 3 Social Determinants of Health Research Center, Health Research Institute, Babol University of Medical Sciences, Babol, Iran; 4 Infertility and Health Reproductive Research Center, Health Research Institute, Babol University of Medical Sciences, Babol, Iran

**Keywords:** Breast cancer, Anxiety, Depression, Quality of life, Mental health

## Abstract

**Background and purpose:**

The global incidence of breast cancer is the highest among all cancers and is the primary reason for cancer-related fatalities. Our study aimed to assess the predictors of quality of life (QOL) and mental health in breast cancer survivors in Northern Iran.

**Methods:**

This cross-sectional study was done on 96 female breast cancer survivors between the ages of 20 and 65 and was based on convenience samples. We gathered information through demographic and fertility data, a QOL survey, and the Hospital Anxiety and Depression Scale (HADS). A significance level of P < 0.05 was set for the analysis.

**Results:**

In this study, results showed that 11.5% of women had the optimal quality of life, 31.3% favorable quality of life, and 57.3% undesirable quality of life. The average HADS score was 20.14 ± 3.07, with anxiety scores of 10.21 ± 2.31 and depression scores of 9.93 ± 1.64. On multiple linear regression, marital relationship and the number of children were predictors of quality of life (β=-17.624, p = 0.023 and β=-7.427, p = 0.016, respectively), as well as the husband’s education and having no history of other cancers in the woman, were the most important predictors of HADS (β = 0.763, p = 0.039 and β=-0.528, p = 0.016, respectively).

**Conclusion:**

It is crucial to provide exceptional care to breast cancer patients during treatment and post-recovery. Emotional and psychological support is a fundamental requirement for their well-being.

## Introduction

Breast cancer is the most cause of cancer-related fatalities among women globally and is responsible for the second-highest number of cancer-related deaths among women in the United States [1]. Despite extensive research efforts in the laboratory, epidemiology, and clinical studies, the incidence rate of breast cancer continues to rise. This disease remains a major source of illness for women, with one in every 20 individuals worldwide and one in every eight people in high-income countries being affected by it [2]. Fewer than 1% of breast cancer cases are diagnosed in men [3]. Breast cancer is the most common type of cancer worldwide, affecting millions of women and, in some cases, men. It is also the leading cause of cancer-related deaths, making early detection and proper treatment crucial for increasing survival rates and improving quality of life for those affected [4].

Studies have shown that the prevalence of mental health disorders among women with breast cancer is high, with 36.7% of women experiencing mood disorders during the early stages of their disease, including 9.6% with major depression and 27.1% with minor depression. Additionally, 14.6% of women were diagnosed with anxiety disorders, with 8.6% in the early stages of the disease and 6% in its advanced stages [5]. The aforementioned factors can have a significant impact on the quality of life (QOL) of individuals affected by breast cancer. The QOL refers to a person’s subjective perception of their well-being and satisfaction with different aspects of their life, which is unique and varies from person to person [6].

The quality of life for individuals coping with chronic illnesses such as cancer encompasses not just physical health, but a sense of well-being that encompasses their ability to perform daily tasks and their satisfaction with managing the disease and any complications arising from its treatments [7]. Additionally, quality of life is a multi-faceted concept that takes into consideration both the positive and negative aspects of an individual’s life, and is evaluated through various dimensions [8]. A study revealed that the quality of life for 40 women who had survived breast cancer and had a history of receiving chemotherapy, as well as 40 women currently undergoing chemotherapy for breast cancer, was low [9].

The process of adapting to cancer begins from the moment of receiving the diagnosis and continues throughout the course of the disease and its treatments [10]. Breast cancer patients often experience significant levels of fatigue and anxiety up to six months post-chemotherapy, which can negatively affect their quality of life. Future research should focus on examining both anxiety and fatigue, particularly the physical and mental subdomains of these symptoms [11]. The quality of life for breast cancer patients has seen significant improvements in recent years, due in part to interventions such as physical activity and psychosocial support. However, there are still many areas of concern, such as the management of pain and lymphedema, sexual function, and future outlook. Despite advancements in psychological assessments of the quality of life in breast cancer patients, there is still much to be understood about what truly matters to patients [12]. Considering the importance of the breast cancer topic, the present study aims to investigate predictors of quality of life and mental health in breast cancer survivors in Northern Iran.

## Method

### Subjects

This cross-sectional study was conducted on a sample of 96 women with breast cancer, aged between 20 and 65 years, who were referred to the Rouhani teaching Hospital in Babol, located in the northern region of Iran, between August 2021 and October 2022. In this study, the sampling method was non-random and convenience samples were used. The inclusion criteria for the study were as follows: Women with breast cancer aged 20–65 years, Iranian nationality and residency in Babol, basic literacy level to complete the questionnaires, willing to sign the informed consent form, no drug addiction, no current medical illness, no speech or hearing impairments that prevented communication with the researcher. On the other hand, the exclusion criteria for the study were: Patient’s unwillingness to continue participating, incompletion of questionnaires, failure to deliver the questionnaires after three weeks of follow-up, hospitalization before the end of the follow-up period, presence or history of mental illnesses, migration, or death. The questionnaires were completed under the guidance of an expert.

### Sample size calculation

The sample size was calculated using a formula that considered a confidence level of 95% and an error of 10% [d = 10%], assuming that p = q = 50%. As a result, the estimated sample size was determined to be 96 participants.

$$n = \frac{{{Z_{1 - }}\alpha _n^2Pq}}{{{d^2}}}$$

Despite the aforementioned formula and the determination of 96 samples for this study, we should note that in regression studies, 10 to 12 samples are sufficient for each variable. Considering that the number of predictive variables in this study is 7, the sample size of 70–84 people was sufficient, and considering the sample loss of 10%, the total number of study samples was considered to be 96 people.

The Rouhani Hospital is the only educational and outpatient referral center for women with breast cancer in Babol City. Therefore, sampling was done on the inclusion and exclusion criteria of the study to complete the sampling. The study included 110 women with breast cancer who were invited to participate. Out of these, 96 individuals completed the demographic/reproductive characteristics and questionnaires, while 14 participants were excluded due to incomplete answers. Thus, the final analysis consisted of 96 women with breast cancer.

### Measurements

The data collection process involved administering demographic-fertility characteristic questionnaires, quality of life (QOL) questionnaires, and the Hospital Anxiety and Depression Scale (HADS) to the participants.

The demographic-fertility characteristics included age, occupation, education level, body mass index (BMI), husband’s age, occupation, and education level, menarche age, duration of marriage, marital status, relationship with spouse, number of pregnancies, history of abortion, number of children, family history of breast cancer, history of other cancers in woman, couple’s relationship and place of residence.

The quality of life of the participants was evaluated using the European Organization for Research and Treatment of Cancer (EORTC QLQ-C30) questionnaire. This questionnaire consisted of 30 questions divided into 5 functional scales (physical, role-playing, emotional, cognitive, and social) and 9 symptom areas (fatigue, nausea and vomiting, pain, dyspnea, sleep disorder, loss of appetite, constipation, diarrhea, and financial problems). The scores of the functional scales and the overall quality of life score ranged from 0 to 100, with higher scores indicating better quality of life and higher function. On the other hand, in the symptom areas, higher scores indicated greater presence of symptoms and problems associated with the disease [13]. The validity and reliability of the QLQ-C30 questionnaire have been tested in several studies and have yielded good results with validity and reliability scores of 76–93% [14, 15].

The HADS questionnaire was used to evaluate mental health. The HADS is a 14-item self-report questionnaire designed to screen for symptoms of depression and anxiety in outpatients. It takes less than five minutes to complete and consists of two separate subscales, one for depression and one for anxiety, each with seven questions. The physical symptoms have been excluded from both subscales to reduce the risk of false positive diagnoses [16]. The reliability of the HADS has been established, with a Cronbach’s alpha of 0.85 for the depression subscale and 0.86 for the anxiety subscale [17]. The Iranian version of the HADS has been confirmed, with a Cronbach’s alpha coefficients of 0.81 for anxiety and 0.78 for depression [18].

### Statistical analysis

The data were analyzed using the Statistical Package for the Social Sciences (SPSS) 25.0. software. Descriptive statistics (mean, standard deviation, frequency distribution table and relative frequency) were used for quantitative and qualitative variables such as demographic-fertility characteristics, components of QOL, and the level of anxiety and depression in women with breast cancer. The Pearson correlation coefficient was used to correlate the overall QOL score with the components of QOL and also the HADS score with the demographic-fertility characteristics. Multiple linear regression analysis was used for predicting factors of QOL and HADS. The significance level of the tests was considered to be P < 0.05.

## Results

Demographic-fertility characteristics are reported in Table 1. The average age of women with breast cancer was 48.86 ± 9.65 and their most educated was diploma (38.5%). Most of them were married (46.9%), housewives (67.7%) and had a family history of breast cancer (70.8%). Also, 32.3% of the husbands of these women were employees. 13.5% of people reported a history of other cancers. Regarding overall QOL, 11.5% had an optimal quality of life, 31.3% a favorable, and 57.3% an undesirable. The functional and symptomatic components of QOL in women are presented in Table 2. In the functional components, a higher score is a sign of a better condition, and the highest score was related to the social component (53.29 ± 21.84). Also, in the symptomatic components, a higher score is a sign of the worse condition of the person, and the highest score was related to the fatigue component (53.64 ± 19.44).

**Table 1:** Demographic-fertility characteristics in population study

Variable	Mean	Standard deviation
Age [year]	48.86	9.65
Husband’s age [year]	54.91	8.43
BMI* [kg/m2]	25.25	3.10
Menarche [year]	12.31	1.24
Gravidity	1.91	2.37
Abortion	0.29	0.82
Number of children	1.57	1.85
Marriage duration	22.32	11.80
**Variable**	**Number**	**Percent**
**Job**		
Employee	31	32.3
housewife	65	67.7
**Marital status**		
Single	27	28.1
married	45	46.9
divorced	13	13.5
the widow	11	11.5
**Education**		
Sub-diploma	17	17.7
Diploma	37	38.5
Academic	35	36.5
**Husband education**		
Sub-diploma	14	14.6
Diploma	16	16.7
Academic	31	32.3
**Residence**		
Urban	69	71.9
Rural	25	26.0
**Family history of breast cancer**		
Yes	68	70.8
No	28	29.2

*BMI: Body mass index

**Table 2:** Functional and symptomatic components of QOL* in women with breast cancer

Components	Mean	Standard deviation
**Functional**		
Physical	38.62	19.77
Role playing	42.53	21.61
Emotional	52.95	19.91
Cognitive	50.69	21.75
Social	53.29	21.84
**Symptoms**		
Fatigue	53.64	19.44
Nausea and vomiting	49.31	22.81
Pain	46.87	19.39
Dyspnea	49.65	22.16
Sleep disorder,	47.91	20.97
Loss of appetite	50.00	21.08
Constipation,	47.22	24.02
Diarrhea	46.87	25.39
Financial problems	52.77	22.51

* QOL: quality of life

**Fig. 1:**
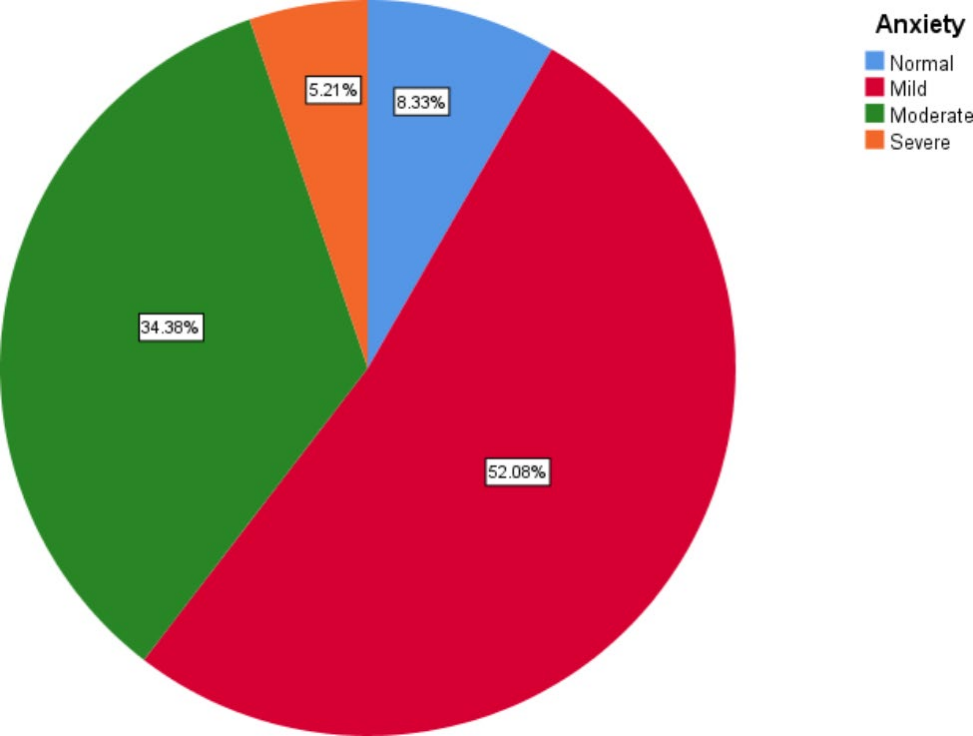
The level of anxiety in women with breast cancer

**Fig. 2:**
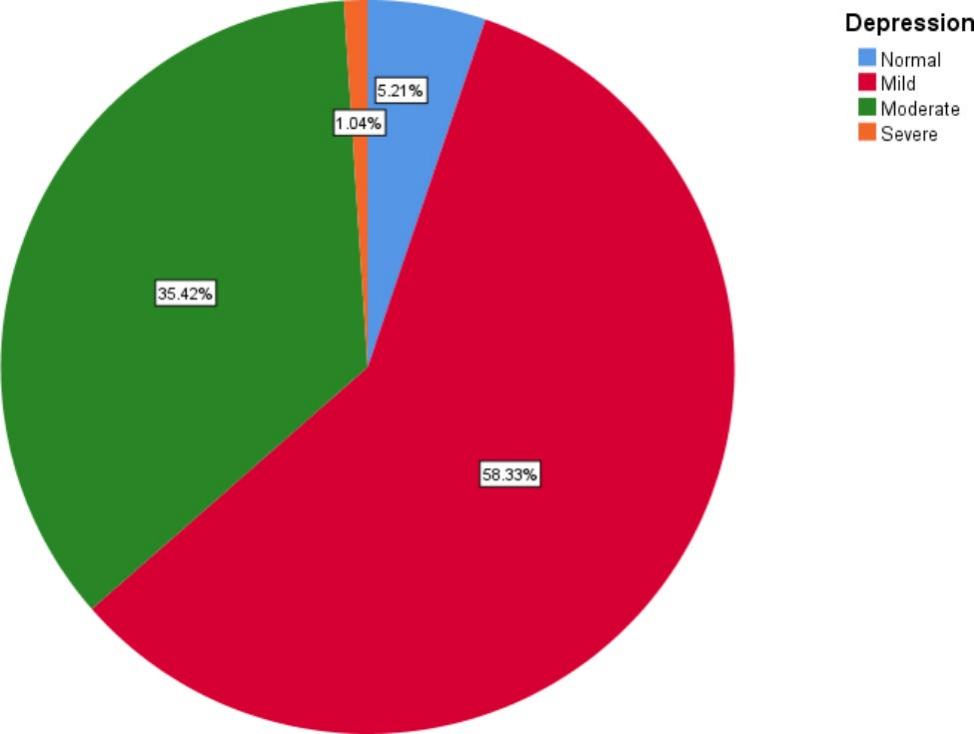
The level of depression in women with breast cancer

The overall HADS score was 20.14 ± 3.07 that the anxiety and depression scores were 10.21 ± 2.31and 9.93 ± 1.64, respectively. Figures 1 and 2 show the degrees of anxiety and depression in women with breast cancer. As can be seen in the figure, the most women had mild anxiety and depression, which were 52.08% and 58.33% respectively. Severe anxiety and depression were present in only 5.21% and 1.04% women, respectively.

Based on the Pearson correlation coefficient, the overall QOL score is correlated with emotional (r=-0.283, p = 0.005), dyspnea (r=-0.200, p = 0.051) and loss of appetite (r=-0.204, p = 0.046) components. Also, the HADS score is correlated with age (r = 0.205, p = 0.045), education (r=-0.299, p = 0.004), husband’s occupation (r = 0.258, p = 0.050), marriage duration (r = 0.254, p = 0.040), sleep disorder (r=-0.207, p = 0.043) and other cancers in women (r=-0.349, p = 0.001).

Table 3 shows factors related to overall QOL in multiple linear regression analysis. This model was able to explain 79% of the total quality of life score by using the variables in the model (R-Square = 0.79), but the variables of this model could not have a statistically significant effect in explaining the total QOL score (p = 0.096). Among the variables of the study, only the variables of marital relations and the number of children had a significant effect on the QOL (β=-17.624, p = 0.023 and β=-7.427, p = 0.016, respectively) and were the most important predictors of QOL. According to these results, for changing one level of status from satisfaction to dissatisfaction, we will have a 64% decrease in the QOL, also for an increase of one child, a 62% decrease in the QOL will be experienced. Table 4 shows multiple linear regression analyses results for related factors with HADS score. This model was able to explain about 35% of the total score of anxiety and depression with the variables included in the model (R-Square = 0.35), however, the effect of these variables to estimate the total score of anxiety and depression was not statistically significant (P = 0.630). Among variables, only the variable of the husband’s education with a 76% increase in anxiety and depression for the increase in husband’s education, as well as the having no history of other cancers in the woman, had a significant decrease of 52% in the reduction of anxiety and depression (β = 0.763, p = 0.039 and β=-0.528, p = 0.016, respectively). These were the most important predictors of HADS.

**Table 3:** Related factors with overall QOL* score in multiple linear regression analysis

Variable	β**	95% CI ***	P- value†
**Constant**		10.05-283.17	0.037
**Age**	0.790	‒0.25-1.82	0.127
**Husband education**	0.488	‒21.57-22.55	0.963
**Number of children**	‒7.427	‒13.26--1.58	0.016
**Marital relations**	‒17.624	‒32.45--2.79	0.023
**Family history of breast cancer**	1.029	‒14.39-16.45	0.889
**HADS** ^****^	‒0.908	‒3.72-1.91	0.505

* QOL: quality of life ** β: Standardized coefficients *** CI: Confidence interval

****HADS: Hospital anxiety and depression scale †The data was assessed using multiple linear regression

**Table 4:** Multiple linear regression analyses results for related factors with HADS* score

Variable	β**	95% CI ***	P- value†
**Constant**		‒1.73-34.75	0.074
**Job**	‒0.001	‒2.936-2.92	0.998
**Husband job**	0.214	‒0.82-2.36	0.332
**Husband’s education**	0.763	0.15–5.77	0.039
**Income**	0.186	‒1.10-2.81	0.889
**Other cancers in woman**	‒0.528	‒7.92- -0.89	0.016
**QOL** ^****^	‒0.032	‒0.05-0.05	0.868

* HADS: Hospital anxiety and depression scale ** β: Standardized coefficients

*** CI: Confidence interval **** QOL: quality of life †The data was assessed using multiple linear regression

## Discussion

In this study, the QOL was optimal in 11.5% of women, relatively favorable in 31.3% of them, and undesirable in 57.3%. In one study, the researchers stated that 42% of women with breast cancer had an unfavorable QOL [19]. In another report, the QOL of women before and after treatment was 69,2 ± 21,1 and 72,0 ± 21,6, respectively [20]. The findings from these studies aligned with our research. Therefore, breast cancer affects the QOL, causes its condition to worsen and suggested that strategies be considered to improve QOL of women.

In this study, in the functional components, the highest score was related to the social component. Also, in the symptomatic components, the highest score was related to the fatigue component. In one study, most people had an unfavorable situation in the field of emotional functioning [19]. In another study, higher mean values were related to cognitive functions, fatigue, insomnia, and pain [21]. Some therapeutic interventions such as music therapy can affect the QOL of cancer patients. In a study, the physical, mental and social dimensions of the music therapy group were increased compared to the control group. Therefore, health and treatment centers should use this method to improve the QOL of cancer patients [22].

In this study, the most women had mild anxiety and depression. Severe anxiety and depression were present in only 5.21% and 1.04% women, respectively. Kokkonen et al. reported that 37% of women with breast cancer had depressive symptoms [23]. In another study, the prevalence of depression and anxiety in patients were 43.4% and 56.2%, respectively [24]. Also, in another study, women had a higher amount of depression and anxiety five to six years than 40 weeks after the diagnosis [25] while in another study, researchers stated that The prevalence of anxiety and depression 5 years after diagnosis was 26.3% and 9.6%, respectively [26]. Patients with cancer tolerate a range of symptoms, including pain and a variegation of physical and psychological distress that affects the quality of life of these patients. Methods such as dignity therapy can modify the quality of life of cancer patients [27].

In this study, the overall QOL score is correlated with emotional, shortness of breath and loss of appetite components. Also, the HADS score is correlated with age, education, husband’s occupation, marriage duration, sleep disorder and own other cancer in the women. In one study, researchers reported that variables of education, age, marital status and financial situation influenced the QOL of women with breast cancer [28]. In another study, lower job status and having children were associated with depression. In addition, surgery type was related to anxiety. Age and comorbidities were predictors for both anxiety and depression [25]. Hajj et al. reported that depression and anxiety levels decrease in people with higher cognitive scores[24].

In this study, only marital relations and the number of children had a significant effect on the QOL and were the most important predictors of QOL. In one study, researchers reported that coping strategies, social support, body image, anxiety, and depression were predictors of QOL [29]. In another study, after adjustment of other variables, women’s job satisfaction was related to QOL in global health status, functional, role, emotional and social scopes in functional scale [30].

The results of this study showed that husband’s education and having no history of other cancers in the women, were the most important predictors of HADS. In one study, comorbidities and age were predictors for anxiety and depression. An increase in depression was more probably when comorbidities and having children. An increase in anxiety was less probably after cancer recurrence [25]. In another study, predictors of anxiety included doubt, younger age, and anxiety at diagnosis. The predictors of depression were pessimism and empathy [26]. Therefore, according to the predictive factors of quality of life and mental health in our study, we should be diligent in managing the excellent relationships of husbands with their sick wives, as well as their all-around support for women despite their education.

### Limitations

One of the limitations of the current research was that the samples were selected from only one hospital. Despite this limitation, the findings of the present study showed the low quality of life of women with breast cancer and depression and anxiety in them. It can also be pointed out that the questionnaires are self-reported, so the participants may not answer the questions accurately for reasons such as impatience, being in a hurry, having an inappropriate mental state.

## Conclusion

In conclusion, considering the results of the study and the unfavorable level of QOL of women with breast cancer, as well as the presence of depression and anxiety in them, it is important to manage these patients in an excellent way during the treatment period and especially during recovery. Psychosocial support for these patients is a basic need. It is suggested that a comparative study with this title be conducted in women with and without breast cancer, or in women with breast cancer and their families.

## Data Availability

All data generated and analyzed during this study are included in this article. Datasets this study are available from the corresponding author upon reasonable request.

## Abbreviations

QOL: Quality of life
HADS: Anxiety and Depression Scale
BMI: Body mass index
EORTC: QLQ-C30 European Organization for Research and Treatment of Cancer
SPSS: Statistical Package for the Social Science
CI: Confidence interval
